# Phytochemical profile, cytotoxic, antioxidant, and allelopathic potentials of aqueous leaf extracts of *Olea europaea*

**DOI:** 10.1002/fsn3.1755

**Published:** 2020-07-19

**Authors:** Amira Zaïri, Sahar Nouir, Amira Zarrouk, Houda Haddad, Améni khélifa, Lotfi Achour

**Affiliations:** ^1^ Laboratory of Biochemistry Faculty of Medicine Sousse Tunisia; ^2^ Laboraory BIOVAL High Institute of Biotechnology Monastir Tunisia

**Keywords:** antioxidant activity., cytotoxicity, infusion, *Olea europaea*, phenolic compounds

## Abstract

Although bioactivities of *Olea europaea* (OE*)* have been widely described, most of them were related to its methanolic extracts or its essential oils, While data related to aqueous extracts still very scarce. Thus, in this study, the phytochemical composition, the antioxidant activity, the cytotoxic potential, and the allelopathic potential of aqueous leaf extracts from two varieties of *Olea europaea* were investigated and compared. High‐performance liquid chromatography (HPLC) was used to identify and quantify the constituents of the tested plants, and spectrophotometric methods to evaluate antioxidant activities. The cytotoxic potential was investigated using murine oligodendrocytes (158N) while germination seeds’ test was used for allelopathic activity. HPLC analysis showed the presence of 10 phenolic compounds in both extracts. Chemlali variety showed the highest antioxidant and allelopathic activities. Regarding the cytotoxicity effect, a significant increase in cell viability was observed with both of our extracts compared to untreated cells. These results confirm that aqueous extracts from OE produce a range of substances with potential antioxidant, antifungal, and allelopathic effects without toxic effects. Thus, they could be used as an alternative of chemical compounds.

## Graphical abstract

Data related to aqueous extracts are relatively rare. Thus, in this study, the phytochemical composition, the antioxidant activity, the cytotoxic potential, and the allelopathic potential of these extracts were investigated and compared.

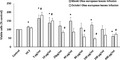

## 1 INTRODUCTION

Since prehistoric times, medicinal plants have been used in traditional medicine practices. Numerous chemical compounds with several functions including defense against herbivorous mammals, fungi, insects, and diseases have been identified (Bishayee & Sethi, 2016; Carocho & Ferreira, 2013). Among these plants is *Olea europaea* L. tree, popularly known as the olive tree, and has a great historical and commercial importance (Nunes, Pimentel, Costa, Alves, & Oliveira, 2016). In fact, *Olea europaea* L. belongs to the Oleaceae family and is one of the important crops and most ancient in the Mediterranean basin which is characterized by long, hot, and dry summers and rainy, mild winters (Romero‐García, Niño, Martínez‐Patiño, Álvarez, & Castro, 2014). However, through a series of morphological, biochemical, and physiological mechanisms, olive tree can cope with the low availability of water in soil in response to lean periods of water in summer (Sofo, Manfreda, Fiorentino, Dichio, & Xiloyannis, 2008).

Olive leaf is one among large amounts of by‐products generated by olive oil production processes (Nunes et al., 2016). This agro‐industrial material has also been widely used in traditional remedies in Mediterranean and European countries (Abd El‐Rahman, 2016). It is generally used as energy biomass or animal feed and considered low‐cost raw materials (Sofo et al., 2008). These aerial parts were also chosen due to their beneficial effects on human health and their important antioxidant potentials (Abd El‐Rahman, 2016; Guex et al., 2019). A study performed by Candar, Demirci, Baran, and Akpınar (2018) showed that cinnamon, black cumin, and olive leaves were the most commonly favored plant products among diabetic patients.

Bioactivities of olive leaf compounds have been reported (El & Karakaya, 2009; Khaliq et al., 2015). Several data showed that they are active against a wide range of microorganisms, have in vitro and in vivo antioxidant activity, antiproliferative effect against cancer and endothelial cells and radioprotective activity (Abd El‐Rahman, 2016; Guex et al., 2019). The potential of olive leaves for the prevention of many human diseases such as hypertension, cardiovascular, and diabetes, has been performed (Orak, Karamac, & Amarowicz, 2019). Recently, the photoprotective potential of olive leaves extract has been also evaluated (da Silva et al., 2019), whereas only minor studies report the bioactivities of the two varieties of leaves of *Olea europaea* sampled in genotypes grown Tunisia (Khlif et al., 2015). To the best of our knowledge, no data are available regarding the cytotoxicity and the allelopathic activities of these varieties.

Due to a high interest on environmental protection and organic agriculture, attention has been focused on allelopathy research. In fact, allelopathy is a biological phenomenon by which an organism produces one or more biochemicals that influence the germination, reproduction, growth, and survival of other organisms (Shengpeng, Zhangshun, Shanyun, Wan, & Han, 2015). Additionally, excessive use of weed herbicides has resulted in a significant emergence of new strains resistant to chemical herbicides which have negative impact on human health and the environment (Mardi & El‐Darier, 2018). Thus, biological control method using natural plants as herbicides will be a better alternative to chemical weedicides. Aqueous olive leaf extracts will be candidate for such assays.

Accordingly, the aims of the current work were to evaluate, for the first time, the photochemical composition, the antiradical potential, as well as the cytotoxicity, and allopathic activities of the aqueous extract of two varieties of *Olea europaea* leaf harvested in the Sahel region of Tunisia Chemlali and Meski.

## 2 MATERIAL AND METHODS

### 2.1 Plant materials

Fresh and healthy leaves of two varieties (Meski and Chemlali) of olive trees (*Olea europaea* L.) were obtained from the Sahel of Tunisia (M’saken, Tunisia). The trees of each variety were grown under the same climatic and soil conditions and were collected in May. The taxonomic identification of all plant samples is identified/authenticated by the forest engineer of Bou‐Hedma Natural Park, and a voucher specimen was deposited at the herbarium of the Laboratory of Medicinal Plants (INAT). Fresh leaves were transported to the laboratory, air‐dried under room temperature, pulverized in a mortar to particles with sizes <0.8 mm, and finely powdered with an electric mill and kept for the extraction process.

### 2.2 Extract preparation: Preparation of the infusion

To prepare infusion extracts, 100 ml of boiled distilled water was added to the sample (5 g) and was stored at room temperature for 5 min. Samples were then filtered under reduced pressure and finally lyophilized. They were re‐dissolved in water to obtain a stock of solution of 1 mg/ml.

### 2.3 Phytochemical analyses and polyphenol content

#### 2.3.1 Total Phenolic Content (TPC)

The TPC of olive leaf extracts was performed using Folin–Ciocalteu's procedure and gallic acid as the standard. 0.25 ml of the sample was combined with 1.25 ml of Folin–Ciocalteu's reagent (diluted ten‐fold), and 1 ml of Na_2_CO_3_ (75 mg/ml). After incubation at 40°C for 40 min, the absorbance of the mixture was measured at 765 nm. All determinations were prepared in triplicate, and quantification was done on the basis of the standard curve of gallic acid. The TPC was expressed as mg gallic acid equivalents (GAE) per g of extract.

#### 2.3.2 Total flavonoids

The amount of flavonoids was determined according to the method of Jia, Tang, and Wu (1999). The extracts (250 μl), distilled water (1,250 μl), and sodium iodide Na_2_NO_2_ (75 μl 5%) were shaken. Then, 150 μl of AlCl_3_ (10%) was added and allowed to stand for 6 min before adding 500 μl of NaOH (1 M) and 250 μl of distilled water. The mixture was left to ambient temperature for 15 min; absorbance was then recorded at 510 nm. The total flavonoid content was expressed in milligrams of catechin equivalent (CE) per gram of samples. Analysis of each sample was carried out in triplicate.

#### 2.3.3 Flavonol content

The content of flavonols was determined by AlCl_3_ method as described by (Miliauskas, Venskutonis, & Van Beek, 2004). Briefly, 500 μl of the plant extract was mixed with 500 μl aluminum trichloride (2%) and 1,500 μl of acetate of sodium (5%). The mixture was shaken and allowed at room temperature in obscurity for 2 hr 30 min. The absorption at 440 nm was then noted. Standard rutin samples were carried out from 0.05 g rutin. All determinations were performed in triplicate. The amount of flavonols in plant extracts was determined in milligrams of rutin equivalents (RUE) per 100 g of extracts.

### 2.4 Identification of active biomolecules of infusions by HPLC

In order to identify and quantify phenolic compounds in the infusion extracts, we restudied them by reversed‐phase high‐performance liquid chromatography (HPLC) analysis by means of a binary gradient elution using Hewlett‐Packard liquid chromatography HPLC (Waldbronn, Allemagne) jointly to an UV–VIS multi‐wavelength detector. For the separation, a Eurospher‐100 C18 reversed‐phase column (250 × 8 mm) was used at room temperature. Acetonitrile (solvent A) and water containing 0.2% sulfuric acid (solvent B) constituted the mobile phase. The flow rate was 0.8 ml/min. The gradient program was in the following way: 15% A/85% B, 0–12 min; 40% A/60% B, 12–14 min; 60% A/40% B, 14–18 min; 80% A/20% B, 18–20 min; 90% A/10% B, 20–24 min; and 100% A, 24–28 min. The injection volume was 20 μl, and peaks were monitored at 280 nm. Samples were filtered through a 0.45‐mm membrane filter before injection.

Identification of peaks was performed by congruent retention times compared with standards. HPLC was used for the quantification of phenolic compounds by comparing peak areas with those of resorcinol used as internal standard. Data were expressed as mg of phenols/100 g of dry weight (DW).

### 2.5 Cytotoxicity analysis by the MTT assay

Murine oligodendrocytes (158N) were used as a cell model to evaluate the cytotoxicity of the infusions extracted from two varieties of *Olea Europaea* leaves (Chemlali and Meski). Dulbecco's modified Eagle medium (DMEM) (Lonza, Amboise, France) supplemented with 5% (v/v) heat‐inactivated fetal bovine serum (Dutscher, Brumath, France) and 1% antibiotics (penicillin, streptomycin) (Dutscher) were used for cell culture. The MTT assay was used to evaluate the effects of treatments on cell viability. MTT salt is reduced to formazan by the mitochondrial enzyme succinate dehydrogenase in the metabolically active cells and was performed on 158N cells as previously described (Nury et al., 2014). Cells were plated in 96‐well culture plates at a density of 1.5 × 10^4^ cells/ well and treated for 24 hr with different concentrations of two varieties of *Olea Europaea* leaf infusion (Chemlali and Meski; 5–400 μg/ml). Vitamin E (α‐tocopherol, 400 µM = 172 µg/ml) was used as positive control. Absorbance of plates was determined at 570 nm with a microplate reader (Mindray, Hamburg, Germany).

### 2.6 Evaluation of biological activities

#### 2.6.1 Evaluation of antioxidant activity

Three different in vitro assays were carried out for the antioxidant activity of our extracts using solutions prepared by serial dilution of stock solution: scavenging effects on DPPH (2, 2‐diphenyl‐1‐picrylhydrazyl) radicals, ferric‐reducing antioxidant power assay (FRAP), and ABTS•+ radical scavenging activity.

##### Determination of DPPH radical scavenging activity

The antiradical activity of olive leaf extracts toward DPPH (1,1‐diphenyl‐2‐picrylhydrazyl radical) was performed according to the method described by Kartal et al with some modifications to adapt the procedure using 96‐well microplates (Kartal et al., 2007). Briefly, 180 μl of various concentrations of extracts (0.009–10 mg/ml) was added to 1,620 μl of DPPH, prepared daily, and kept in the dark when not used. The absorbance was measured at 517 nm. The scavenging activity was expressed as IC50, and the extract dose needed to induce a 50% inhibition. A lower IC50 value refers to a higher antioxidant activity of plant extract. The ability to scavenge the DDPH radical was determined using the following equation: [(A_DPPH_^‐^ A_S_)/A_DPPH_]*100, where A_S_ is the absorbance of the solution containing the sample at 517 nm, and A_DPPH_ is the absorbance of the DPPH solution.

##### ABTS^+^ scavenging activity

ABTS^+^ scavenging activity was mixed with 88.0 μl of a 140 mM potassium per‐sulfate (K_2_S_2_O_8_) solution overnight in the dark. Prior to the assay, a dilution of the ABTS with ethanol to an initial absorbance of about 0.700 (ratio of 1:88) at 734 nm was conducted. Free radical scavenging activity was then performed by mixing 975 µl ml of diluted ABTS with 25 μl of sample at concentrations ranging from 0.039 to 10 µg/ml. Absorbance was measured after 20 min. All tests were carried out in triplicate. Ascorbic acid was used as a positive control.

##### Determination of ferric‐reducing antioxidant power (FRAP)

Ferric‐reducing antioxidant power of infusion extract experiment was conducted according to Li et al. (2007) Method. Samples were mixed with 2.5 ml sodium phosphate buffer (pH 6.6) and 1% potassium ferricyanide (2.5 ml). After incubation at 50°C for 20 min, 10% trichloroacetic acid (2.5 ml) was added to 2.5 ml of distilled water and 0.1 ml of ferric chloride (0.1%) and the mixture was centrifuged. The absorbance was measured at 700 nm. Results were expressed on (EC_50_) (Li et al., 2007).

#### 2.6.2 Allelopathic bioassay

Germination test was used to study the possible allelopathic effects of two different studied varieties of *Olea europaea* (Meski and Chemlali) on seeds of *Triticum aestivum* and *Linum usitatissimum*. Healthy and uniform seeds were sterilized with 95% chloric acid and washed with distilled water more than two times. Twenty seeds were placed with suitable amount of different concentrations of the infusions (0.25, 0.5 and 1 mg/ml) in sterilized Petri dishes provided with two layers of filter paper. Three replicates were prepared from each concentration, and distilled water (0%) was used as a control. To avoid reduction of moisture content of the blotting paper, equal volume of distilled water was added to the Petri dishes. Seeds incubated at 25ºC and were observed daily and considered germinated when the radical was approximately 1 mm long or more. The percentage of germination is calculated according to the following formula:

% G = (NGG/ NTG) *100

% G: Percent of germination (%)

% IG: Percent of inhibition's germination: 100‐ % G = (NGG/NTG) *100%

NGG: Number of seeds germinated in the presence of water or extract

NTG: Total number of seeds

### 2.7 Statistical analysis

The experiment results were statistically analyzed by SPSS Statistics for Windows version 21,0, and Student's *t* test and Duncan's multiple range test were used.

## 3 RESULTS AND DISCUSSION

### 3.1 In vitro Toxicity of Extracts against Murine Oligodendrocytes (158N)

As far as we know, few reports are available on the cytotoxicity of plant extracts before proceeding to their biological activities. It is worth mentioning that the present study was also the first endeavor to evaluate the cytotoxicity of *Olea europaea* extracts. Cell viability in the presence of *Olea europaea* leaf infusions was evaluated via the MTT test, which quantifies the enzymatic activity of succinate dehydrogenase. In the presence of Meski *Olea europaea* leaf infusion (5–200 μg/mL), significant increase in cell viability was observed compared to untreated cells (control), with the exception of 400 μg/mL concentration for which the cell viability decreased to 60%. In addition, similar effects were seen in the presence of Chemlali *Olea europaea* leaf infusion at 5 and 10 µg/ml only. Reduction in cell viability was remarked with 400 µg/ml of Chemlali infusion. Both leaf infusions showed better effect on cell viability compared to Vit E treated cells.

Since *Olea europaea* leaf infusions had a high amount of polyphenols, they could be used as an alternative for Vit E, known as the most potent antioxidant molecule. Indeed, Vit E was able to counteract 7‐ketocholesterol‐induced cell death as well as the associated mitochondrial dysfunctions in the same cell line used in this study (Nury et al., 2018). On the basis of this experiment, which has been frequently used to assess the cytoprotective effects of different compounds, the *Olea europaea* leaf infusions were used in this study. In fact, several studies reported the roles of olive leaves and their polyphenolic constituents on redox homeostasis, attenuation of necrotic and apoptotic cell death in the presence of different ROS inducers, such as H_2_O_2_ (Cumaoğlu et al., 2011 **).**

### 3.2 Total polyphenol, flavonoid, and flavonol contents

The screening of phytochemical of the tested plants showed that the presence of polyphenols, flavonoids, and flavonols in high concentration was observed in both Chemlali and octobri extracts. The infusion extracts of the leaves of the Chemlali cultivar had the highest total phenols (545.90 mg GAE/g), flavonoids (604.94 mg CE/g), and flavonols (109.25 mg RE/g ext) amounts, followed by the Meski cultivars (Table 1).

**TABLE 1:** Results of total phenolic, flavonoid, and flavonol contents of Chemlali and Meski olive leaf (*Olea europaea* L.) extracts

	Total phenols (mg GAE/g ext)	Flavonoids (mg Cat/g ext)	Flavonols (mg RE/g ext)
Meski	480.34 ± 1.36	506.4 ± 1.91	72.95 ± 0.05
Chemlali	546.06 ± 2.55**	605.25 ± 3.17**	109.35 ± 0.17**

Note

Values are mean ± standard deviation.

** Indicate significant difference between values at *p* < .001 level (Student's *t* test).

The quantity of total phenolic compounds in our two tested *plants* was higher than the one revealed in other local Olea. In fact, Edziri et al. (2019) reported that TPC and flavonoid amounts of methanolic extract of *Olea europaea cultivar Meski* obtained from the northwest of Tunisia are 34.55 mg GAE/g and 5.34 mg CE/g, respectively. In light of our knowledge, no data were also provided concerning the amount of TPC or flavonoids or flavonols in aqueous extracts in Tunisia. Nevertheless, the TPC of olive leaf of Tunisian genotypes in the present study is higher than that noted for Algerian cultivars. In a recent study, Debib et al, 2016, showed that Algerian methanolic extract of Chemlali olive leaves had the highest total phenolic content (21.47 ± 0.05 mg GAE/g dried matter) followed by aqueous extract (10.5 ± 1.23 mg GAE/g dried matter). In other scientific reports, it was shown that leaf extracts of *Olea europaea* are rich in phenolic contents. For example, in the present study, the TPC of olive leaf of Tunisian genotypes is similar to that noted for Turkish (230.15–241.60 mg GAE/g dry leaves) and Italian (40.9–66.6) cultivars (Orak et al., 2019 **).**

As it is known, the amount of TPC of plant extracts varies from one herb to another in different areas in the world. This difference can be explained by many factors such as genetic origins, environmental climate effect (short‐growing season, hot temperature, dryness, high solar exposure), soil composition, and the type of solvent used through the extraction protocol (Edziri et al., 2019; Zairi et al., 2018). So it appears that phenol content showed noticeable variations with plant growth. In fact, according to the life cycle of olive leaves, the phenol storage in the leaves is probably a time‐dependent regulated process. In addition, Ozcan et al showed that phenol contents and fatty acid composition of the olive oils may differ according to olive variety (Özcan et al., 2019).

### 3.3 Identification of active biomolecules by HPLC

**FIGURE 1:**
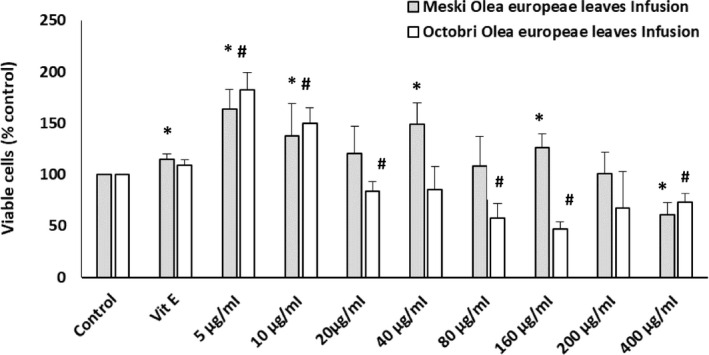
Effect of Olea europaea leaf infusions on 158N cell growth and/or mitochondrial activity. 158N cells were incubated in the presence of infusions (5–400 μg/ml; 24 hr). “*” and “#” indicate a significant difference between untreated cells (control) and Meski or Octobri Olea europaea leaf infusion‐treated cells (Mann–Whitney test; *p* < .05)

**FIGURE 2:**
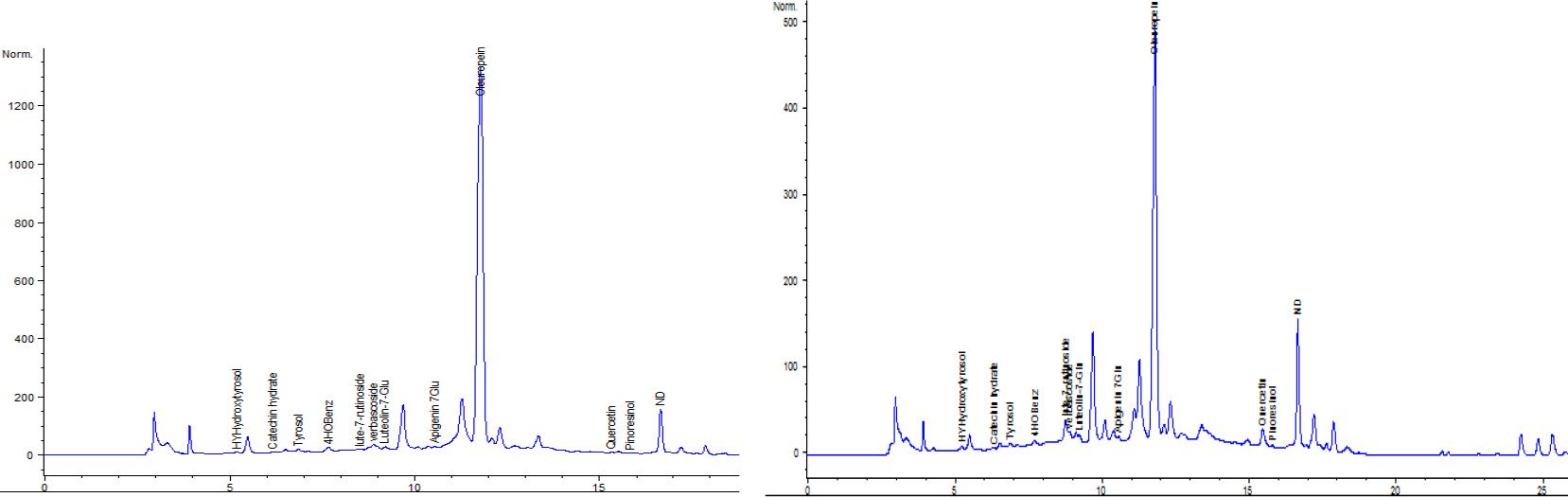
HPLC chromatograms from leaf aqueous extracts of *Olea europaea* (Chemleli and Meski)

Figure 1 showed the HPLC separation of the phenolic compounds of olive leaf extract. It permitted the identification of ten compounds including hydroxytyrosol, 4HOBenz and tyrosol (substituted phenol), catechin hydrate (flavan‐3‐ols), lute‐7‐rutinoside (Flavonoids), verbascoside and oleuropein (oleuropeosides), luteolin‐7‐Glu; apigenin 7Glu, and quercetin (flavones). As described in Table 1, oleuropein and verbascoside, a glycosylated conjugate of caffeic acid and hydroxytyrosol, were the predominant phenolic compounds in both Meski and Chemlali extracts. We also noted that a small difference in phenolic composition was observed in the tested cultivars. The above cited compounds were previously determined in olive leaves but in other extract types (Edziri et al., 2019; Meirinhos et al., 2005; Sahin & Bilgin, 2012). It is for the first time that the aqueous extracts, especially infusion, were analyzed for their phenolic composition (Figure 2).

The content in the examined plants was comparable to the results of other researchers working on some *Olea europaea* species in other extract. The work of Ezdiri et al. used samples of *Olea europaea* from north and south of Tunisia after extraction with methanol. The authors identified oleuropein as the most abundant phenolic component common to all the two test cultivars with the highest amount was observed in the Meski (Edziri et al., 2019). These results are also in agreement with (Brahmi, Mechri, Dhibi, & Hammami, 2012), who showed that the olive organs from the southern cultivars (Chemlali) had the highest level of oleuropein. Based on these results, we can conclude that polyphenol compounds are more abundant in methanolic than aqueous extracts.

Furthermore, as far as we know, few studies are conducted to determine the phenolic composition of aqueous extracts of *Olea europaea* in Tunisia. The evaluation of these extracts is very limited, only few plants have been performed to date, and no data have been described before to determine the polyphenol composition of decoction or infusion extracts. Almost all the available studies in the literature reported mostly on methanolic extracts or essential oils (Brahmi et al., 2012; Edziri et al., 2019; Khlif et al., 2015).

The differences seen in the phenolic profiles of extracts from various origins might be related to the growing and the environmental conditions (soil composition, climate, altitude, rainfall). They can directly interfere with the content of chemical components (e.g., phenolic compounds) and as a consequence in their therapeutic effects (Edziri et al., 2019). In our work, some of these factors were removed: Olive trees were grown under the soil conditions and same climatic and leaves’ collection was done within one month. These results show that each extraction method enhanced the recovery of specific phenolic groups with different performance.

### 3.4 Assessment of biological activities

#### 3.4.1 Evaluation of the antioxidant activity in vitro

Food industry and agricultural researchers use frequently DPPH, FRAP, and ABTS assays to evaluate the antioxidant activity of plants. These free radicals give an idea about the potential of the extract compounds to delay oxidative cell damages. The chelating activity IC_50_ obtained using three different methods is also shown in Table 2. All tested extracts showed a high reducing power in a concentration‐dependent manner (Table 2). The lower the IC50 values, the higher the chelating capacity of the plant extract. Thus, the highest radical scavenging effectiveness was observed with aqueous extracts of *Chemlali* cultivar followed by Meski cultivar, thereby attesting its higher capacity in eliminating the formed reactive oxygen species (Table 3).

**TABLE 2:** Amount of phenolic compounds in % of extracts of *Olea europaea* (Chemlali and Meski) leaves identified by HPLC

Compounds	Chemlali		Meski	
	Retention time (min)	Quantity in %	Retention time (min)	Quantity in %
Hydroxytyrosol	5.195	0.172	5.221	0.281
Catechin hydrate	6.177	0.133	6.318	0.233
Tyrosol	6.849	0.713	6.875	1.074
4HOBenz	7.670	1.203	7.697	2.630
Lute‐7‐rutinoside	8.526	0.902	8.753	5.014
Verbascoside	8.890	6.881	8.891	6.035
Luteolin‐7‐Glu	9.204	2.738	9.224	4.347
Apigenin 7Glu	10.551	0.482	10.558	1.060
Oleuropein	11.788	85.672	11.797	74.512
Quercetin	15.323	0.133	15.468	2.091

**TABLE 3:** In vitro antioxidant activities of the infusions olive leaves obtained using two different varieties Meski and Chemlali

	DPPH	ABTS	FRAP
Meski	0.19 ± 0.01	0.78 ± 0.001	1.13 ± 0.01
Chemlali	0.13 ± 0.002*	0.30 ± 0.001**	0.93 ± 0.001*

Note

Values are mean ± standard deviation.

* indicates significant difference between values at *p* < .05 level.

** indicates significant difference between values at *p* < .001 level (Student's *t* test).

All the extracts had a promising scavenging effect attesting the presence of some compounds that are electron donors and convert free radicals into more stable products.

Furthermore, the evaluation of the aqueous extracts of *Olea europaea* is very limited, no local plants have been performed to date, and no data have been described before to demonstrate the scavenging ability of infusion extracts. The majority of studies available in the literature reported mostly antioxidant activity of plant's essential oil or methanolic extracts. As an example, Brahmi et al studied the antioxidant activity of different cultivars (cv) of Tunisian olive. They showed that methanolic extracts of the cv. “chetoui” had greater antioxidant potential on DPPH radicals when compared to those reported for fruits and leaves of cv. “chemlali” (Brahmi et al., 2012). The results reported in this experiment are completely in accordance with a study already published by Edziri et al showing the potent antioxidant activity of four methanolic extracts of Tunisian olive cultivars. They reported that, among all tested cultivars, Meski methanol extracts have the lesser activity (47.3%). These data could be a good reason to develop and evaluate other type of extracts such aqueous extracts (Edziri et al., 2019).

Furthermore, previous studies regarding different *Olea europaea* extracts from other countries have been investigated for their antioxidant activity. As an example, Cheurfa et al studied the antioxidant activity of Algerian *Olea europaea*. They reported that ethanol extract of *O. europaea* leaves showed considerably higher (*p* < .05) DPPH radical scavenging activity than aqueous extract (IC_50_ 92.04 mg/ml) (Cheurfa et al., 2019). These data also suggest that the antioxidant activity of our extracts is significantly higher than those cited above, since only 0.301 mg/ml of chemlali cultivar was sufficient to induce the same effect. Although there have been plenty of reports on DPPH or ABTS radical scavenging activity of *Olea europaea* species, the lack of the utilization of aqueous extracts makes it difficult to compare their antioxidant strength.

These high antioxidant activities could be related to the presence of some bioactive compounds such as their high amount of total phenolic content (TPC) and flavonoids, especially may be due to oleuropein, a well‐known antioxidant derivative (Orak et al., 2019). Indeed, findings of the HPLC analysis showed the richness of oleuropein, in aqueous extracts of Chemlali cultivar which could explain its highest antioxidant activity as compared to Meski cultivars.

Interestingly, some recent studies showed that other phenolic compounds can be involved in the antioxidant activity of olive leaves. For example, Alsharari, Al‐Rejaie, Abuohashish, Ahmed, and Hafez (2016) revealed that rutin previously identified in *O. europaea* leaves attenuated hepatotoxicity caused by oxidative stress in high‐cholesterol‐diet‐fed rats. Besides, previous study suggested that luteolin could provide protective effects against the progression of diabetes‐induced cardiac dysfunction by reducing oxidative stress (Wang et al., 2009). Hydroxytyrosol and oleuropein were reported to have antioxidant properties in vivo (Cheurfa et al., 2019; Jemai, Bouaziz, Fki, El Feki, & Sayadi, 2008). Thus, we can conclude that antioxidant activity depends on extraction drying methods and cultivars and is affected by chemical‐composition in the tested plants (Đorđević, Sarić‐Krsmanović, & Umiljendićb, 2019; Edziri et al., 2019).

#### 3.4.2 Evaluation of allelopathic effect in vitro

The impact of allelopathy on composition and the structure of biological communities is relatively uninvestigated in olive extracts (Thiébaut, Thouvenot, & Rodríguez‐Pérez, 2018). Thus, the aim of this study is to assess whether the aqueous leaf extracts (infusion) of the *O. europaea* had an allelopathic effect on seed germination of *T aestivum and Linum usitatissimum*. The results reported in Table 4 confirmed that *Olea europaea* leaves contain a naturally occurring allelopathic substance in dose‐dependent manner.

**TABLE 4:** Allelopathic effect of infusion leaves of Olea europaea (Chemlali and Meski) on *Triticum aestivum* and *Linum usitatissimum*

Concentrations	% of germination inhibition (*Triticum aestivum*)	% of germination inhibition (*Linum usitatissimum*)
Chemlali		
1%	59.00 ± 1.00^a^	69.00 ± 1.00^a^
0.5%	37.50 ± 1.00^c^	58.33 ± 1.53^b^
0.25%	32.67 ± 0.58^d^	52.33 ± 0.58^c^
Meski		
1%	44.00 ± 1.00^b^	40.67 ± 0.58^d^
0.5%	32.33 ± 2.52^d^	38.67 ± 0.58^d^
0.25%	16.33 ± 1.53^e^	35.67 ± 0.58^e^

Note

Different superscript letters in the same row indicate significant difference between values at *p* < .05 level (Tukey's test), and values are mean ± standard deviation

Noticeably, germination percentage displayed a gradual decrease with the increase of all *O. europaea* aqueous extracts. As can be seen in Table 4, over 80% of the seeds germinated in control. However, response to olive leaves was high; thus, germination of *Triticum aestivum* and *Linum usitatissimum* seed was inhibited by our extracts. Seeds irrigated by aqueous extract obtained from Chemlali cultivar exhibited the highest percentage of inhibition of the germination (70%) followed by Meski leaf extract (35%) compared to control. These data are in disagreement with a recent study conducted by (Al‐Samarai, Mahdi, & Al‐Hilali, 2018), who showed that aqueous extracts of olive leaves from Iraq produced the lowest rate of inhibition (23%) of *Cyperus rotundus L*. The lack of the utilization of aqueous extracts makes it difficult to compare their allelopathic strength.

The inhibitory potential of allelopathy is complex, and different groups of chemicals can be involved such as phenolic compounds, terpenoids, flavonoids, alkaloids, amino acids, steroids, and carbohydrates (Al‐Samarai et al., 2018). It can be due to the congenital effects of plant containment in soluble substances in water and to biotic and the fluctuations of abiotic parameters, such as climatic conditions (Petrussa et al., 2013), the presence of herbivores and/or pathogens (Gatti et al., 2014; Silva, Overbeck, & Soares, 2014) and stage in the life history of the plant (Lombardo, Mjelde, Källqvist, & Brettum, 2013; Santonja, Le Rouzic, & Thiébaut, 2018; Thiébaut et al., 2018). In recent data, it was reported that the inhibitory activity may be attributed to the accumulation of glucosinolate in the plant parts, especially the leaves (Al‐Samarai et al., 2018). In this case, infusion samples might secrete allelopathic compounds to eliminate and destroy other plant species. In nature, four main ways are used to release allelochemicals, for instance, leaching, decomposition, volatilization, and exudation.

## 4 CONCLUSION

The results of the present data clearly showed that the use of aqueous extracts such as infusion could be a better and more promising source of antioxidant and allopathic agents than ethanolic or methanolic extracts. Devoid of toxicity and being rich in phenolic compounds, either phenolic acids or flavonoids, aqueous extracts could be benefic for health to overcome oxidative stress and reactive species production. Additionally, they could be a solution for inhibiting plant invasion. They could inhibit seedling growth, making them useful for eco‐friendly, biocontrol program management. However, further experiments are warranted in order to purify, explore the mechanisms of action involved, and elucidate the structure of active compounds for the development of a new class of biological metabolites.

## CONFLICT OF INTERESTS

The authors declare that there is no conflict of interest.
